# Cancer resection rates, socioeconomic deprivation, and geographical access to surgery among urban, suburban, and rural populations across Canada

**DOI:** 10.1371/journal.pone.0240444

**Published:** 2020-10-14

**Authors:** Blake Byron Walker, Nadine Schuurman, Chuck K Wen, Saad Shakeel, Laura Schneider, Christian Finley

**Affiliations:** 1 Institut für Geographie, Friedrich-Alexander-Universität Erlangen-Nürnberg, Erlangen, Germany; 2 Dept. of Geography, Simon Fraser University, Burnaby, British Columbia, Canada; 3 University of Toronto, Toronto, Ontario, Canada; 4 Division of Thoracic Surgery, McMaster University, Hamilton, Ontario, Canada; Massachusetts General Hospital, UNITED STATES

## Abstract

High-risk cancer resection surgeries are increasingly being performed at fewer, more specialised, and higher-volume institutions across Canada. The resulting increase in travel time for patients to obtain treatment may be exacerbated by socioeconomic barriers to access. Focussing on five high-risk surgery types (oesophageal, ovarian/fallopian, liver, lung, and pancreatic cancers), this study examines socioeconomic trends in age-adjusted resection rates and travel time to surgery location for urban, suburban, and rural populations across Canada, excluding Québec, from 2004 to 2012. Significant differences in age-adjusted resection rates were observed between urban (14.9 per 100 000 person-years [95% CI: 12.2, 17.6]), suburban (40.7 [40.1, 41.2]), and rural (32.7 [29.6, 35.9]) populations, with higher rates in suburban and rural areas throughout the study period for all cancer types. Resection rates did not differ between the highest (Q1) and lowest (Q5) socioeconomic strata (Q1: 13.3 [12.2, 14.4]; Q5: 12.0 [10.7, 13.4]), with significantly higher rates among middle-SES patients (Q2: 27.3 [25.6, 29.0]; Q3: 39.6 [37.4, 41.8]; Q4: 37.5 [35.3, 39.7]). Travel times to treatment were consistently higher among the most socioeconomically deprived patients, most notably in suburban and rural areas. The results suggest that the conventional inclusion of suburbs with urban areas in health research may obfuscate important trends for public health policy and programmes.

## Background

Cancers are the leading cause of death in Canada, with 197 000 new diagnoses and 78 000 deaths per year [1]. However, improvements in tumour detection and treatment continue to extend patient survival; nearly two-thirds of patients survive at least five years post-diagnosis [1].

Surgical tumour resection is an effective treatment modality, but comorbidities, high-risk surgical procedures, and other complicating factors often demand a high level of operative specialisation and medical facilities. To meet these demands, many high-risk resection procedures are increasingly being performed at fewer, more specialised centres in major Canadian cities. This process of regionalisation to high-volume institutions has been shown to reduce overall patient mortality rates and post-operative length of stay [2–4], including in Canada [5]. However, the average travel time for a patient to reach their location of surgery has increased as a result [5].

Patient access describes an individual’s ability to interface with medical systems in order to receive treatment [6]. Access therefore relies both on a patient’s ability to physically reach a treatment centre (spatial access) and their social and economic position, e.g., language barriers or financial inability to travel (socioeconomic access) [7, 8]. Spatial access has been shown to be a significant predictor of tumour stage at diagnosis [9], choice of treatment [10–12], and mortality [13], with greater travel time to treatment observed for rural patients [14]. Socioeconomic status is also a known predictor of cancer survival [13, 15] and surgery rates [16, 17], and though the causative pathway is unclear, the burden of long-distance travel to treatment may be exasperated by a lack of social and financial resources [5, 14]. Few studies to date have considered a combination of both spatial and socioeconomic measures of access, with several notable exceptions [13, 18, 19], though the identification of socioeconomically deprived and spatially isolated patient populations has significant implications for cancer control policy and health equity.

The binary categorisation of study populations into urban and rural patients has highlighted important differences in choice and outcome of cancer treatments [14, 20–22]. However, there is a growing body of evidence suggesting that suburban neighbourhoods are becoming increasingly socioeconomically deprived in North America [23, 24], with many of the deprivation-associated health risks and outcomes [25–28]. Our recent work differentiates suburban neighbourhoods from their urban and rural counterparts, documenting elevated oral cancer incidence rates, high levels of health-related socioeconomic deprivation [29], and low access to treatment centres.

Identifying geographical and socioeconomic disparities in access to cancer treatment is important for improving health equity. The regionalisation of high-risk cancer resection surgeries during the study period may increase disparities in access among socioeconomically deprived rural and suburban populations, who already face additional barriers to spatial and socioeconomic access. This study therefore sought to identify socioeconomic patterns in cancer resection rates between urban, suburban, and rural populations, and to examine socioeconomic differences in urban, suburban, and rural patients’ geographical access to surgery.

## Data and methods

Approvals for this study were obtained through both the Simon Fraser University Research Ethics Board (2014s0154) and the Hamilton Integrated Research Ethics Board (13–790). All patient records were anonymized prior to receipt and patient consent was therefore not required.

Patient data were obtained from the Canadian Institutes of Health Information (CIHI) Discharge Abstract Database, comprising all surgeries reported by hospitals in Canada, excluding Québec. Data may be requested directly from CIHI (https://www.cihi.ca/sites/default/files/document/data-requests-research-infosheet-2020-en-web-rev_1.pdf). We included all patients discharged from 1 April 2004 to 31 March 2012 (the latest date for which complete data are available) following a tumour resection surgery for the sites corresponding to the ICD-10 diagnosis codes for oesophageal, ovarian/fallopian, liver, lung, and pancreatic cancers, with the surgical intervention codes shown in Table 1. Patient data comprised the following fields: age, sex, home postal FSA (the first three digits of a Canadian postal code), institution where the surgery was performed, date of discharge, and all ICD-10 diagnosis and intervention codes.

**Table 1:** ICD-10 diagnosis and intervention codes selected for analysis.

Tumour Site	ICD-10 Diagnosis Codes	ICD-10 Intervention Codes
**Oesophageal**	C150-155, C158-159, D377	1NA89DB, 1NA89FA, 1NA91DB, 1NA91FA, 1NA88DCXXG, 1NA88FCXXG, 1NA87FB, 1NA87FC, 1NA87DC, 1NA87DD, 1NA87EY, 1NA87EZ, 1NA87QG, 1NA87QH, 1NA88LBXXG, 1NA88QFXXG, 1NA89LB, 1NA89QF, 1NA90LBXXG, 1NA90LBXXG, 1NA90QFXXG, 1NA91LB, 1NA91QF, 1NA92LBXXF, 1NA92LBXXG, 1NA92QFXXG, 1NA87LD, 1NA87LE, 1NA87QC, 1NA87QD
**Ovarian/Fallopian**	C560-561, C569, C5700-5701, C5709, C571-574, C578, D391	1RB87DA, 1RB89DA, 1RD89DA, 1RB87LA, 1RB89LA, 1RD89LA, 1RD89RA, 1RB87RA, 1RB89RA, 1RF87DA, 1RF89DA, 1RF87LA, 1RF89LA, 1RF87RA, 1RF89RA, 1RM87BAGX, 1RM89CA, 1RM87CAGX, 1RM91CA, 1RM89AA, 1RM87DAGX, 1RM89DA, 1RM87DAAG, 1RM91AA, 1RM91DA, 1RM89LA, 1RM87LAGX, 1RM91LA, 1OT87LA, 1OT87DA
**Liver**	C220-224, C227, C229, C787, D376	1OA87DA, 1OA87LA, 1OA87LAAZ
**Lung**	C3400-3401, C3409-3411, C3419, C342, C3430-3431, C3439, C3480, C3489, C3490-3491, C3499, C390, C398-399, D381	1GR87DA, 1GR87PN, 1GT87DA, 1GR87NW, 1GR87QB, 1GT87NW, 1GT87QB, 1GR89DA, 1GR89NW, 1GR89QB, 1GR91NW, 1GR91NWXXA, 1GR91NWXXG, 1GR91NWXXN, 1GR91QB, 1GR91QBXXA, 1GR91QBXXF, 1GR91QBXXG, 1GR91QBXXN, 1GR91QBXXQ, 1GT89NW, 1GT89QB, 1GT91NW, 1GT91NWXXF, 1GT91NWXXG, 1GT91NWXXN, 1GT91NWXXQ, 1GT91QB, 1GT91QBXXF, 1GT91QBXXG, 1GT91QBXXN, 1GT91QBXXQ, 1GT89DA
**Pancreatic**	C250-254, C257-259	1OJ87LA, 1OJ87VK, 1OJ87VC, 1OJ87DA, 1OK87LA, 1OK87VZ, 1OK87WA, 1OK87XN, 1OK91LA, 1OK91XN, 1OK89LA, 1OJ89LA, 1OJ89VZ

This study used a geographical data linkage methodology to assign each patient’s neighbourhood type (urban/suburban/rural) and socioeconomic deprivation score, described as follows. Population data from the 2006 census were obtained from Statistics Canada for every dissemination area (DA; n = 39445; each DA has 400–700 residents) in the study area. Each DA was categorised by its neighbourhood type using the multivariable method by Gordon and Janzen [30], based on a validated combination of Organization for Economic Cooperation and Development and Statistics Canada definitions [31] and transportation variables, as outlined in Table 2. This literature-based definition was heuristically selected as it best represents the qualitative characteristics of a suburban neighbourhood in Canada [30].

**Table 2:** Neighbourhood type definitions, from Gordon and Janzen [30].

Neighbourhood Type	Definition
**Rural**	Population density < 150 persons/km^2^
**Urban Core**	In a Statistics Canada Census Metropolitan Area (CMA); and % of population that commutes via active transit modes (walking, cycling, etc.) > = 1.5 times the CMA average
**Suburban**	Neither rural or urban

A socioeconomic deprivation score was also calculated for every DA using the Vancouver Area Neighbourhood Deprivation Index, a multivariate weighted index for health-related socioeconomic disadvantage based on seven material and social variables from the 2006 census: average income, secondary school completion, university degree attainment, lone-parent families, home ownership, employment ratio, and unemployment rate [32]. The resulting scores were then used to derive deprivation quintiles, where Q1 is the least deprived (highest socioeconomic status) and Q5 is the most deprived (lowest socioeconomic status).

Patient data were available only at the FSA scale (there are 1635 in Canada and the median FSA population is 17433). Approximately the size of a neighbourhood in urban areas and a county in rural regions, FSAs are geographically larger than DAs, containing an average of 24 DAs. As a result, DA-level neighbourhood types and deprivation scores were aggregated to derive averages for each FSA in the study area as follows: the DA-level neighbourhood types were coded as 1 = urban, 2 = suburban, 3 = rural; these values were then population-weighted by the number of adults (18+ years) in each DA. The mean population-weighted neighbourhood type for all DAs within an FSA was then calculated to derive an index value ranging from 1 to 3. This value was rounded to the nearest integer to assign a neighbourhood type to an FSA. Similarly, each FSA was assigned the mean population-weighted deprivation score of all its DAs within, and each FSA was classified by its socioeconomic deprivation quintile, based on deprivation scores for the entire Canadian population. The resulting FSA-level neighbourhood type and deprivation score was then linked to each patient in the dataset by patient home FSA code.

Resection rates provide a useful indicator of patient access [33, 34]. Age- and sex-specific resection rates were calculated for each cancer type (tumour site), socioeconomic deprivation quintile, neighbourhood type, and 3-year interval (2004–2006, 2007–2009, 2010–2012). Three-year intervals were selected following sensitivity analysis, as they provide adequate temporal resolution while maintaining statistical power for the subsequent tests. These rates were all adjusted to 5-year age groups corresponding to the 2006 Canadian standard population using the direct method, with 95% CI estimated using the binomial approximation method used by the International Agency for Research on Cancer [35]. For ovarian/fallopian cancers, only the female denominators and standard populations were used. Differences in adjusted resection rates between socioeconomic groups were assessed using a two-sample binomial proportions test and significance was defined using a threshold p-value of 0.05, selected for consistency with the literature.

Prior to our receipt of the patient data, each patient’s travel time from their place of residence to the location where their surgery took place was calculated in a secure facility using geographical information systems. (Our team did not have access to patient residential information.) The shortest-time route was calculated along a road and ferry network dataset comprising intersections, speed limits, and other transportation network features, but excluding air travel. The resulting travel times were tabulated for each neighbourhood type and socioeconomic deprivation quintile, and their means with 95% CI were calculated. All statistical and geographical analyses were conducted using SPSS v.21 and ArcGIS v.10.3.

## Results

Complete data were acquired for 61 127 patients. As shown in Table 3, over 61% of cases were female, due largely to the inclusion of ovarian cancers. Nearly three-quarters of all patients were found to reside in predominantly suburban areas, compared to 65% of the Canadian population. Similarly, only 6.9% of patients resided in urban areas, less than half the population proportion (16%). A lower proportion of patients were observed in the most and least socioeconomically deprived quintiles, a pattern consistent for all five cancer types. Lung cancers comprised half of all cases, and despite prevalence only among the female population, ovarian/fallopian cancers comprised over a quarter of the total number of cases examined in this study.

**Table 3:** Number of patients by cancer site, with proportions of total cases for each categorical variable and number of unique institutions at which a surgery was performed.

		Oesoph.	Ovarian	Liver	Lung	Pancreatic	All Sites	Per Cent
**Sex**	**Female**	566	16575	3220	15487	1880	**37728**	61.7%
	**Male**	2187	0	4898	14304	2010	**23399**	38.3%
**Neighbourhood Type**	**Urban**	131	1286	565	1991	270	**4243**	6.9%
	**Suburban**	2003	12279	6230	21581	2921	**45014**	73.6%
	**Rural**	619	3010	1323	6219	699	**11870**	19.4%
**Socioeconomic Deprivation Quintile**	**Q1 (high SES)**	242	2120	1031	2563	491	**6447**	10.5%
	**Q2**	557	3798	1997	5866	876	**13094**	21.4%
	**Q3**	914	4969	2484	9102	1226	**18695**	30.6%
	**Q4**	832	4470	2068	9280	1000	**17650**	28.9%
	**Q5 (low SES)**	208	1218	538	2980	297	**5241**	8.6%
**No. of Surgeries**	**2004–2006**	866	5387	2159	9191	1094	**18697**	
	**2007–2009**	883	5353	2564	10030	1233	**20063**	
	**2010–2012**	1004	5835	3395	10570	1563	**22367**	
**No. of Institutions**	**2004–2006**	67	197	85	69	67		
	**2007–2009**	53	194	70	64	60		
	**2010–2012**	46	184	68	55	57		
	**Total**	**2753**	**16575**	**8118**	**29791**	**3890**	**61127**	
	**Per Cent**	4.5%	27.1%	13.3%	48.7%	6.4%		

Half of the hospitals included in this study were in suburban neighbourhoods, and 40% were in dense urban cores. The remaining 10% of hospitals were in rural areas. Deprivation scores of the neighbourhoods in which hospitals were located exhibited a nearly identical mean and variance as the Canada-wide distribution of deprivation scores. A decrease in the number of hospitals at which procedures were performed is observed for all five tumour sites in Table 3. Across the study period, one-third of all hospitals in the study featured an average of one or fewer resections per year (very low volume), whilst 73% of all hospitals in the study had an average of one or fewer resections per month (low volume). One-fifth of hospitals in the study held between 1 and 10 resections per month (medium volume), whilst approximately 7% of resections were held in high-volume centres (more than 10 resections per month). The hospitals with the highest volume of resections were Toronto General (5142), Vancouver General (4636), Victoria Hospital in London, Ontario (3137) and Foothills Medical Centre in Calgary, Alberta (3060). The proportion of all resections taking place in high-volume centres increased during the study period from 5.9% to 11.5%. A more detailed examination of these trends can be found in Finley et al. [5].

**Fig 1:**
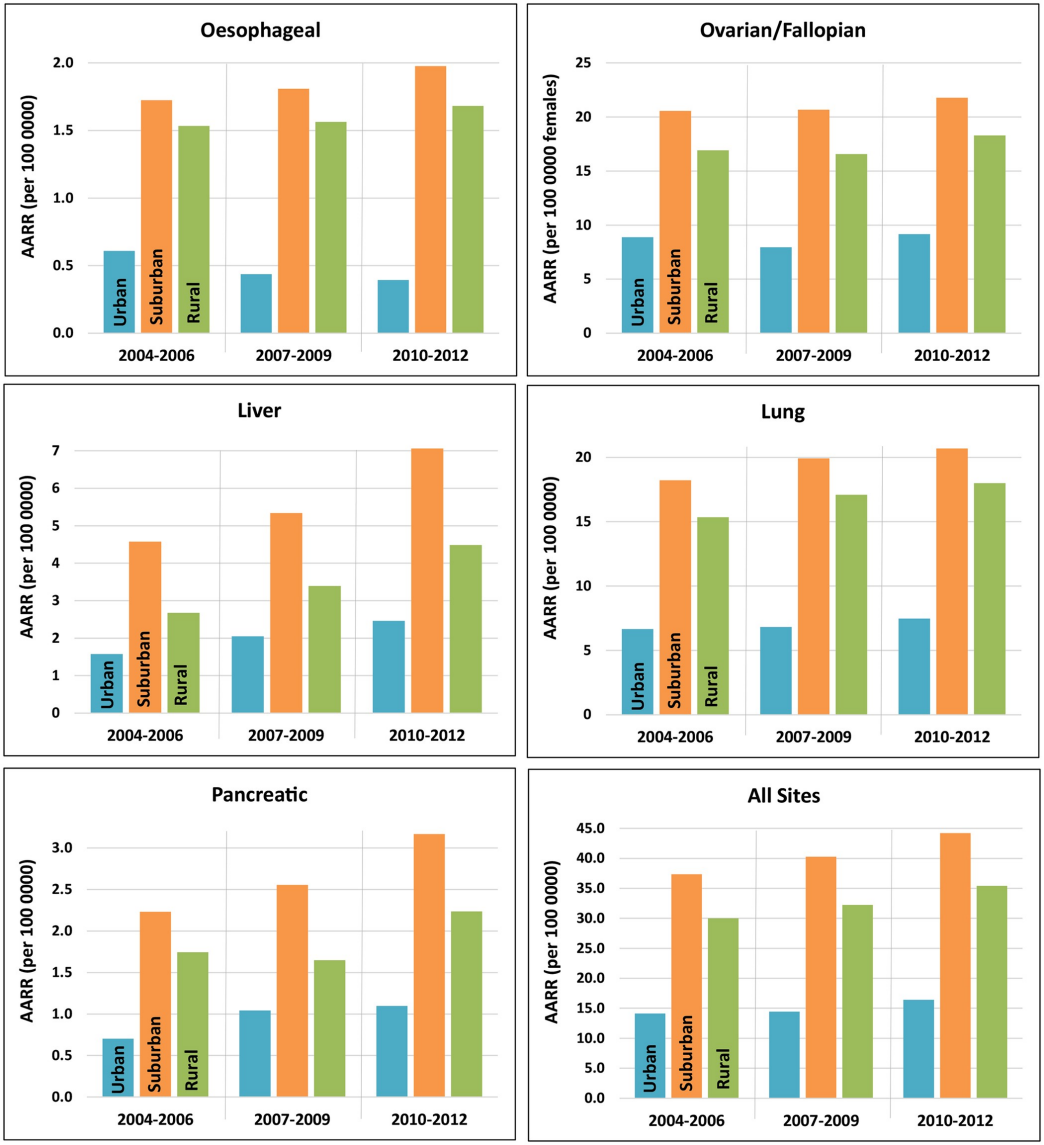
Age- and sex-adjusted resection rates by tumour site and socioeconomic deprivation quintile, in 3-year intervals from 2004–2012.

When categorised by socioeconomic deprivation quintile, age- and sex-adjusted resection rates were approximately equal for patients among the most deprived (Q5: 12.0 per 100 000 person-years [10.7, 13.4]) and the least deprived (Q1: 13.3 [12.2, 14.4]) quintiles of the population, with higher rates among middle-SES patients (Q2: 27.3 [25.6, 29.0]; Q3: 39.6 [37.4, 41.8]; Q4: 37.5 [35.3, 39.7]). When stratified by cancer site and 3-year period, this pattern was found to be consistent for all five cancer types (Fig 1). The socioeconomic gradient is particularly pronounced for lung cancers.

**Fig 2:**
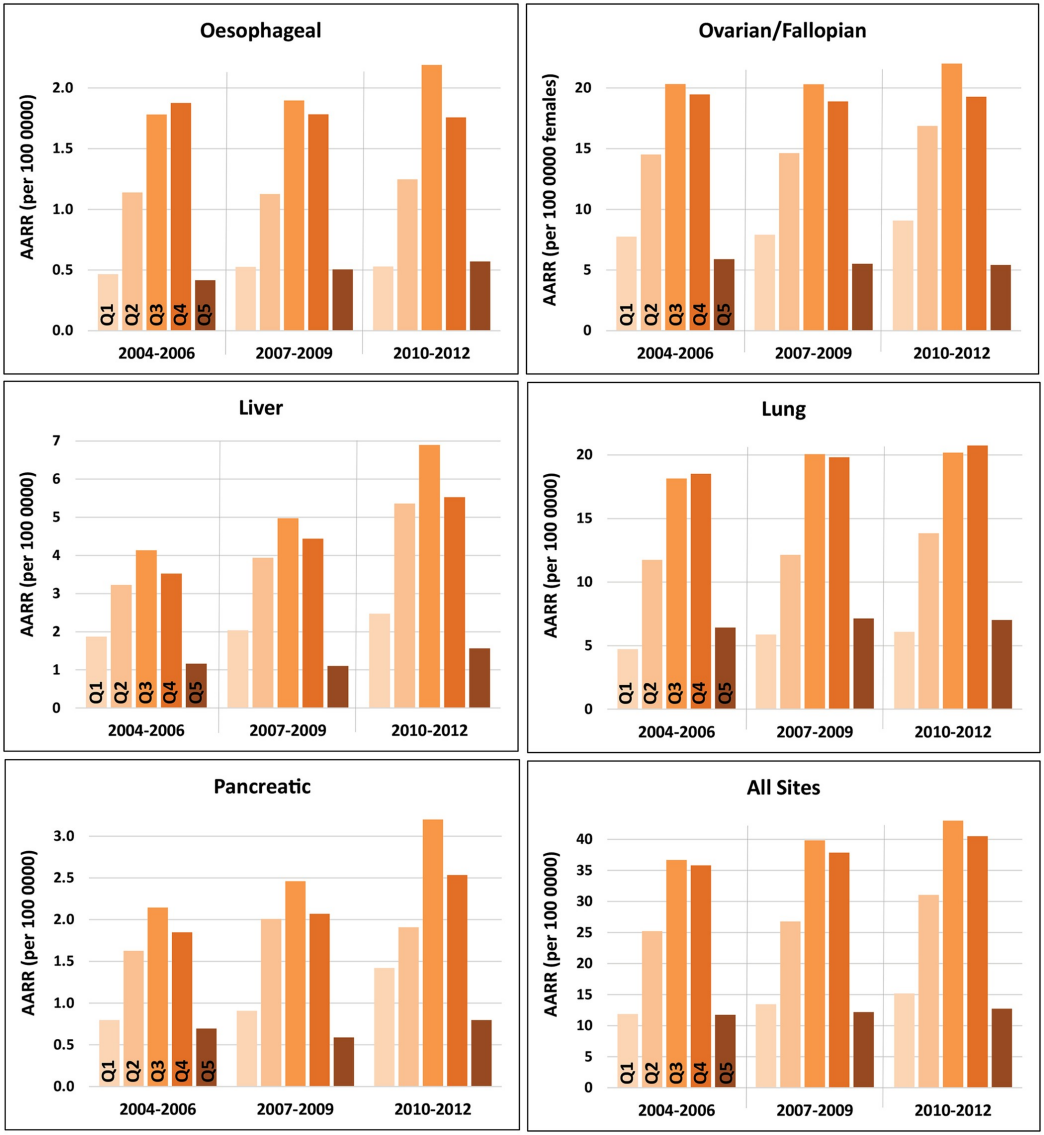
Age- and sex-adjusted resection rates for five cancer types, by neighbourhood type, in three-year intervals from 2004–2012.

Large differences in resection rates were observed between urban (14.9 [12.2, 17.6]), suburban (40.7 [40.1, 41.2]), and rural (32.7 [29.6, 35.9]) populations, with consistently higher rates in suburban and rural areas throughout the study period. When categorised by neighbourhood type, the highest adjusted rates were observed in suburban areas for all cancer types across the entire study period (Fig 2). The urban-suburban disparity is particularly pronounced for oesophageal cancers, for which a decline in urban rates was observed, in contrast to increasing suburban and rural rates. Large suburban-rural differences were observed for liver and pancreatic cancers but are relatively minor for other tumour sites.

**Fig 3:**
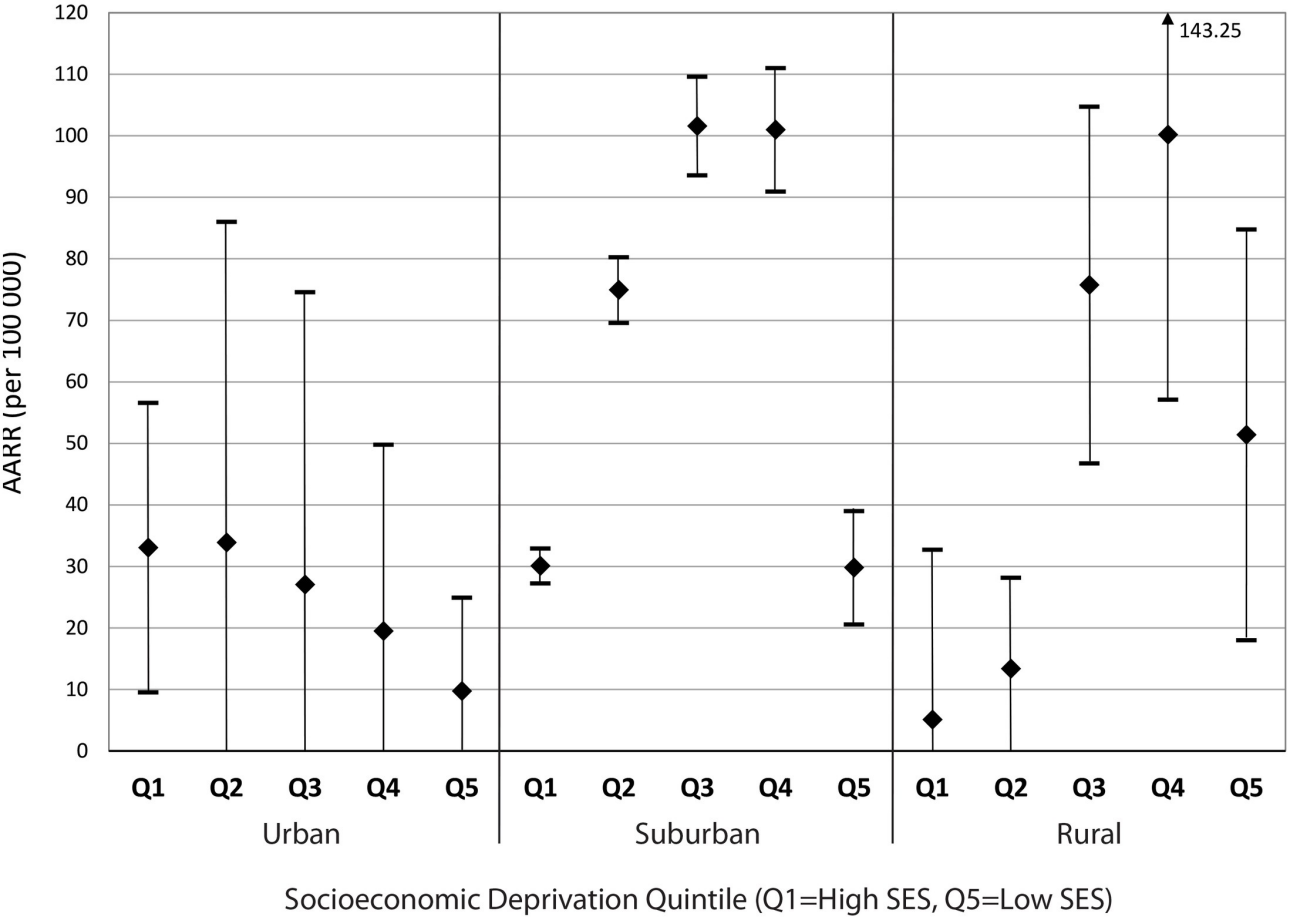
Age- and sex-adjusted resection rates per 100 000 person-years, with 95% CI, by neighbourhood type and socioeconomic deprivation quintile (Q5 = patients among the 20% most deprived adults in the Canadian population).

When pooled, resection rates for all five cancers types exhibit highly significant differences between socioeconomic groups in both suburban and rural neighbourhoods (Fig 3). A low number of urban cases resulted in inadequate statistical power to reliably test for significant differences. However, it is observed that resection rates follow a socioeconomic gradient: increasing deprivation corresponds with decreasing resection rates. Suburban and rural resection rates exhibit similar distributions across the socioeconomic gradient, except for the most affluent groups, which featured relatively lower resection rates.

**Fig 4:**
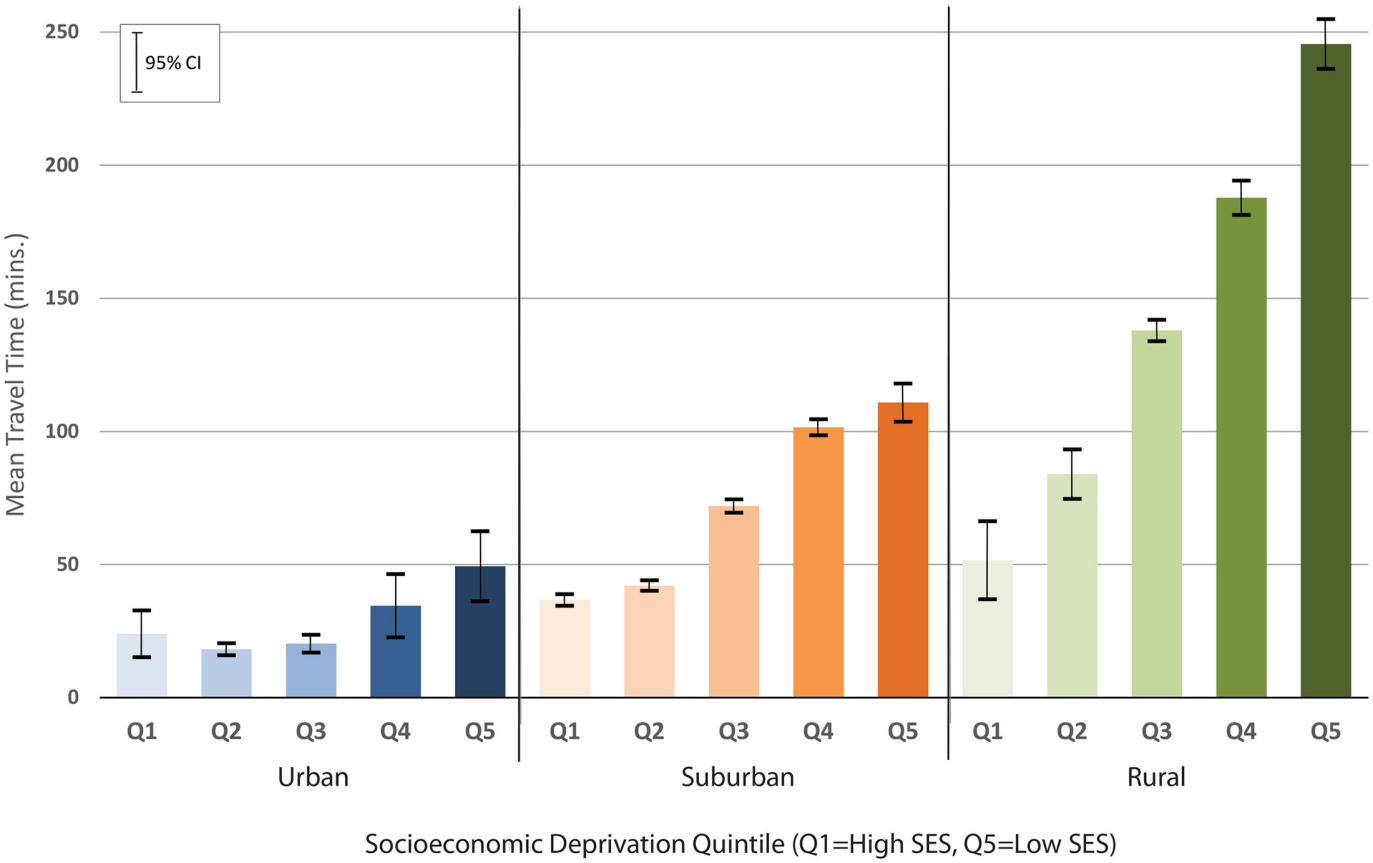
Mean patient travel time from resection surgery location.

Mean patient travel times for all five cancer types exhibit a socioeconomic gradient such that the greatest burden of spatial access is experienced by patients in the most socioeconomically deprived quintiles of the population, as shown in Fig 4. This pattern is relatively weak among urban patients, with more distinct differences observed for those living in suburban and rural neighbourhoods. Significant differences in incidence between urban, suburban, and rural populations are detected only among middle-SES groups.

## Discussion

This study identifies a significant difference in resection rates and their relationship with socioeconomic deprivation between urban and suburban areas. For most tumour sites, previous studies have observed consistently higher incidence and treatment rates among urban populations compared to rural [20, 22, 36]. Our results demonstrate the inverse for oesophageal, ovarian/fallopian, liver, lung, and pancreatic cancers. However, this difference may be due to our categorisation scheme; whereas previous studies have classified urban areas such that they include suburban areas, we distinguish suburban neighbourhoods from their urban cores. Interestingly, suburban resection rates and travel times, and their relationships to socioeconomic deprivation, are more similar to rural areas than urban. These elevated rates among rural patients may also reflect poor access to detailed counselling against high-risk surgeries and potential substitutes alternative treatment modalities (such as radiotherapy for the treatment of advanced lung cancers).

The observed relationship between travel time and socioeconomic deprivation is consistent with our previous study evaluating potential spatial access to cancer treatment centres for head and neck cancer patients [29]. By conducting this study with actual patient data, we are herein able to confirm a concentration of high travel times among the most socioeconomically deprived patient populations. This pattern reflects the socioeconomic geography of Canada, where the mean deprivation score is highest in rural areas (0.32) and lowest in suburban neighbourhoods (-0.19), with urban residents experiencing relatively average socioeconomic deprivation (-0.01). Regardless, our findings confirm that the longest travel times to surgery are experienced amongst populations with higher average socioeconomic deprivation. Previous studies have explored this hypothesis for urban/rural categories, but our results indicate that more geographically refined analysis with the inclusion of suburban areas is needed [17, 21].

A previously published reported highlights that increases in resection rates throughout the study period are coincident with decreasing incidence rates for most cancer types [1], which may indicate that a higher proportion of patients are receiving surgical treatment. However, it is possible that these trends reflect higher overall prevalence. Additionally, the observed socioeconomic differences in resection rates may be attributable by differences in population prevalence and stage at diagnosis, although our data and methods were not able to analyse this hypothesis. Individuals living in urban centres often are diagnosed at an earlier stage due to improved access [9], which may account for some of the variance in resection rates observed in this study. However, oncologist and patient decision-making is highly complex and varies on a case-by-case basis, dependent on tumour stage/grade, patient comorbidities and history, availability of facilities and personnel, personal preference, ability to travel for treatment, etc. All of these factors are geographically and socioeconomically relevant and merit further analysis.

A series of three citizen focus groups conducted in Edmonton, Hamilton, and Charlottetown underscored concerns about patients living in rural/remote regions [5]. Despite a general preference for regionalisation of cancer treatment, participants highlighted a need for local patient support networks for pre- and post-operative care. The majority of participants also indicated a willingness to travel great distances to receive high-quality treatment, although travel costs, a desire to receive care close to home, and family commitments were all significant concerns. The importance of spatial access may also be a significant factor at a finer regional scale, which may partially explain the observed differences between urban and suburban resection rates.

While our overall study population was relatively large (N = 61 227), the number of patients for some categories was insufficient to enable statistically sound inference. However, it must be noted that these data represent a population of patients, not a sample; the reported rates therefore reflect the most rigorous estimates available for the study area. To estimate local socioeconomic deprivation scores, the median DA score was taken for each FSA. This aggregation results in a loss of resolution for the socioeconomic data, and may have impacted our findings. An FSA contains an average of 24 DAs, with an average within-FSA variance of 0.49 deprivation units, indicating reasonable socioeconomic heterogeneity within FSAs; however, variances were higher in rural FSAs than in urban or suburban, indicating a higher probability of error in our deprivation estimates for rural patients. The next phase of study will attempt to use higher-resolution patient data (e.g., complete 6-digit postal codes) to better detect and quantify these effects. Another important limitation in this study is the method used to calculate travel times. This model does not include air travel, which may have caused an overestimation of travel times for patients residing in Northern Canada. That data from Québec were not available is also a considerable limitation in this study; improved interoperability of datasets between administrative bodies is recommended to facilitate future epidemiological research.

The most notable limitation of this study is the study period, which ends in 2012. While we are able to document evidence of socioeconomic gradients in travel time to resection surgery, it is unclear whether these patterns have changed more recently. The next phase of study will seek to use more geographically precise and recent data to investigate these patterns in greater detail. Additionally, there have been changes in treatment modalities since 2012 that may affect patient decisions regarding travel, and may also exhibit a socioeconomic gradient, for example, neoadjuvant therapy prior to resection from oesophageal sites. Future analysis should include other treatments as potential covariates for more detailed modelling of the associations between socioeconomic deprivation, resections, and travel time.

## Conclusion

Contrary to previous studies, we observed the highest resection rates among patients in the central quintiles of socioeconomic deprivation. This may be due to our differentiation of suburban patients from their urban counterparts, which yielded patterns of resection rates, travel times, and socioeconomic deprivation more similar to rural patients than urban ones. We conclude that further research addressing population patterns of access to cancer treatment, including both demand and utilisation, would benefit from this differentiation. This novel categorisation has implications for cancer control policy, specifically when devising strategies to target vulnerable communities in the most deprived and geographically isolated communities across Canada. So while regionalisation has led to improved outcomes overall, particular focus on rural and socioeconomically deprived patient populations is necessary to maintain health equity across the country.
